# HSV-1 Cytoplasmic Envelopment and Egress

**DOI:** 10.3390/ijms21175969

**Published:** 2020-08-19

**Authors:** Imran Ahmad, Duncan W. Wilson

**Affiliations:** 1 Department of Developmental and Molecular Biology, Albert Einstein College of Medicine, 1300 Morris Park Avenue, Bronx, NY 10461, USA; imran.ahmad@einsteinmed.org; 2 Dominick P. Purpura Department of Neuroscience, Albert Einstein College of Medicine, 1300 Morris Park Avenue, Bronx, NY 10461, USA

**Keywords:** herpes simplex virus, HSV-1, envelopment, ESCRT, microtubules, sorting

## Abstract

Herpes simplex virus type 1 (HSV-1) is a structurally complex enveloped dsDNA virus that has evolved to replicate in human neurons and epithelia. Viral gene expression, DNA replication, capsid assembly, and genome packaging take place in the infected cell nucleus, which mature nucleocapsids exit by envelopment at the inner nuclear membrane then de-envelopment into the cytoplasm. Once in the cytoplasm, capsids travel along microtubules to reach, dock, and envelope at cytoplasmic organelles. This generates mature infectious HSV-1 particles that must then be sorted to the termini of sensory neurons, or to epithelial cell junctions, for spread to uninfected cells. The focus of this review is upon our current understanding of the viral and cellular molecular machinery that enables HSV-1 to travel within infected cells during egress and to manipulate cellular organelles to construct its envelope.

## 1. Introduction

Herpes simplex virus types 1 and 2 (HSV-1 and HSV-2) and varicella zoster virus (VZV) are human pathogens of the subfamily *Alphaherpesvirinae* that replicate in peripheral tissues then invade the nervous system to establish latency [1,2,3,4,5]. In the case of HSV-1 the latent viral genome persists as a circular dsDNA episome in the nuclei of neurons of the trigeminal ganglia (TG) [3,4,6]. Upon reactivation [1], viral gene expression and DNA replication lead to assembly of progeny viral particles that exit the nucleus and undergo anterograde transport along axonal microtubules (MTs) to reach the nerve terminal and infect adjacent peripheral epithelial tissues [4,5,7,8] such as oral and anogenital mucosa and ocular epithelia [1,3,9]. Replication in neuronal and epithelial cells requires HSV-1 capsid trafficking and envelopment in the cytoplasm, and delivery of the resulting organelle-associated enveloped virions (OEVs) to the nerve terminal, cell surface, or cell–cell junctions for subsequent transmission to uninfected cells. The focus of this review is upon the mechanisms of cytoplasmic envelopment, intracellular trafficking, and sorting of HSV-1 capsids and enveloped virions. In some instances, where it helps illuminate less well understood aspects of HSV-1 biology, we refer to data obtained for the related swine alphaherpesvirus pseudorabies virus (PRV, suid alphaherpesvirus 1) [4,6,7,10].

**Figure 1:**
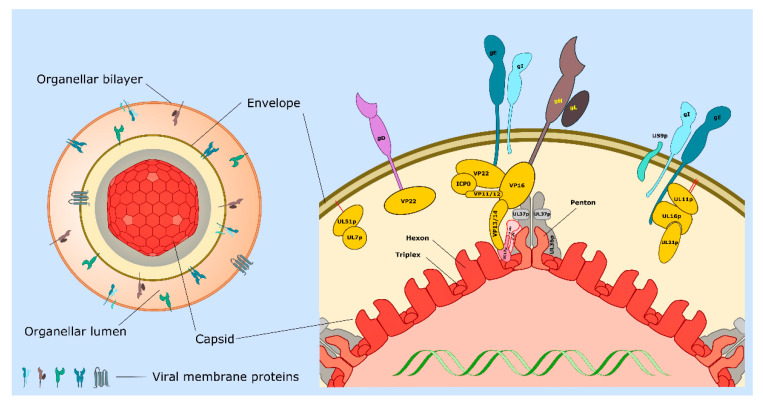
Structure of the cytoplasmic herpes simplex virus type 1 (HSV-1) particle during egress. Left: The organelle-associated enveloped virion (OEV). The icosahedral capsid (with red hexons and pale red pentons) is bound to inner tegument proteins including UL36p and UL37p (gray layer), which provide the foundation for attachment of outer tegument (yellow). Tegument connects the capsid to the surrounding envelope (dark brown line) containing numerous single and multiple membrane-spanning envelope proteins. The mature virion resides within the lumen (orange) of the organelle utilized for envelopment. Prior to capsid envelopment the lipid bilayer of the bounding organelle must contain all of the membrane proteins that become incorporated into the mature envelope. This figure assumes that these envelope proteins persist in the bounding membrane of the OEV, though in most cases this is unknown. Right: Expanded view of a region of the capsid shell (red), inner- (gray), and outer- (yellow) tegument proteins and the envelope (dark brown bilayer). VP5 hexons and pentons are colored as in left-hand particle, and hexons, pentons, and triplexes are indicated. At the penton vertices each copy of VP5 is connected to a UL36p dimer and to adjacent triplexes via a UL17p/(UL25p)_2_ complex (for clarity the figure shows only two copies of VP5 at the vertex, and one copy of UL17p/(UL25p)_2_). Envelope proteins gD, gE/gI, gH/gL, and US9p (Table 1) are shown imbedded in the envelope bilayer such that their cytoplasmic tails project inward to connect with tegument proteins. Red lines indicate lipid anchors stabilizing the interaction of UL11p and UL51p with the envelope. Some proteins (for example gE and VP22) participate in multiple interactions within the tegument. These are drawn as separate complexes for clarity, but we do not mean to imply that these associations are necessarily exclusive of one another. See Table 1 and text for more details.

The mature HSV-1 particle is composed of about 40 structural proteins distributed between three distinct layers (Figure 1, left). The ~125 nm diameter icosahedral capsid enclosing a single copy of the linear dsDNA genome encoding approximately 80 open reading frames, a lipid bilayer envelope derived from an organelle of the host cell which contains multiple virally encoded membrane proteins, and a complex multi-subunit protein layer termed tegument that lies between the capsid and envelope. Many of the protein–protein interactions that support the assembly and function of the HSV-1 particle are summarized in the right half of Figure 1, and in Table 1. The structure and composition of HSV-1 particles have also recently been reviewed in detail [10,11,12,13,14].

## 2. Emergence of Non-Enveloped Capsids from the Nucleus and Recruitment of Inner Tegument 

Following reactivation from latency, the HSV-1 genome is replicated and packaged into preassembled viral procapsids [1,11,14]. DNA-containing nucleocapsids then bud into the inner nuclear membrane and pinch off as primary enveloped virions into the perinuclear space, a process catalyzed by the UL31p/UL34p nuclear export complex [15,16]. The fusion of this primary envelope with the outer nuclear membrane delivers the capsid into the cytoplasm [15,16,17,18,19]. The shell of the mature HSV-1 icosahedral capsid is composed of the major capsid protein UL19p (VP5) which assembles into capsomeres; pentamers (pentons) at the vertices and hexamers (hexons) on the icosahedral faces (Figure 1) [11,20,21]. The hexons and pentons are connected by a triplex linker formed from one copy of UL38p (VP19C) and two copies of UL18p (VP23) [11,20]. Prior to egress from the nucleus, or soon after arrival in the cytoplasm (see below for more details), the capsid becomes associated with inner components of the tegument layer (Figure 1). Tegument is composed of more than a dozen distinct virally encoded proteins and several host proteins (Section 3.6) [10,11,22,23] that participate in motor recruitment and MT-directed transport of capsids, docking of capsids to organelles, cytoplasmic envelopment to form the mature virion [11,12,13] (detailed in Section 3), and perform multiple tasks following entry and infection of new host cells [1,2]. The most capsid-proximal “inner” tegument is largely composed of UL36p [11,24], its binding partner UL37p [11,25], and the serine/threonine kinase US3p [26,27,28] (Figure 1, Table 1). With a mass of ~330 kDa, UL36p is the largest protein encoded by the *Herpesviridae.* Two copies of UL36p decorate each of the five molecules of VP5 at the capsid vertex, bound via a dimer of UL25p and one copy of UL17p, which serve to attach UL36p to VP5 and adjacent triplexes [11,21,24,29,30,31,32] (Figure 1). UL17p and UL25p, together with a portion of UL36p, form the capsid vertex-specific component (CVSC), a region of density visible in capsid cryoEM studies lying over the penton-proximal triplexes [11,14,20,21,24].

UL36p provides a foundation for the recruitment of “outer” tegument; it binds the outer tegument protein UL48p (VP16), which in turn connects to UL46p (VP11/12), UL47p (VP13/14), UL49p (VP22), and membrane-imbedded envelope proteins [11,33,34,35,36,37] (Figure 1, Table 1, and see Section 3.6 for more details, reviewed in [11,38]). UL36p also directly binds its inner tegument partner UL37p [33,39,40,41], a ~120 kDa protein with an amino terminal portion structurally similar to cellular multisubunit tethering complexes (MTCs) that facilitate docking of transport vesicles to target membranes during their intracellular trafficking [42,43] (Section 3.5). An essential and conformationally flexible carboxy-terminal region is thought to permit UL37p to associate with several additional viral and cellular binding partners [25,44] as detailed in Section 3.2. UL36p and UL37p participate in multiple events during HSV-1 assembly and trafficking, described in greater detail below (Section 3). 

It is not clear whether inner tegument proteins are added to the HSV-1 capsid while it resides in the nucleoplasm, or only after emergence into the cytosol. Both UL36p and UL37p localize at least in part to the interior of the nucleus [101,102,103], but immunoblot analysis has led to differing conclusions concerning whether UL36p and UL37p are bound to nuclear capsids [53,104,105,106]. More recently, studies of HSV-1 capsids extensively purified from nuclei and subjected to both immunoblotting and immunoelectron microscopy concluded that nuclear capsids were associated with UL36p, UL37p, US3p [27], and the infected cell polypeptides 0 and 4 (ICP0 and ICP4), immediate-early proteins that regulate gene expression and transactivation of early and late viral genes [27,107,108]. Similar conclusions were reached by the same group using an in vitro capsid nuclear-egress assay; in this assay system the only source of inner tegument proteins was the nucleoplasm, and capsids emerging from the nucleus were found to be associated with UL36p, UL37p, ICP0, and ICP4 [27]. Nevertheless, other studies reported that ICP0 and ICP4 are attached to HSV-1 capsids at a post-nuclear step [109,110]. 

Work with PRV may shed some light on the confusion regarding the location and timing of UL36p addition. Although most studies agree that UL36p is attached to the PRV capsid in the cytoplasm [11,111,112,113,114], a carboxy-terminal fragment of PRV UL36p has been observed to translocate to the nucleus, associate with nuclear capsids, and enhance capsid export to the cytoplasm [115]. Once the PRV capsid reaches the cytoplasm, the UL36p-derived fragment departs and is replaced by full-length UL36p [115]. If a similar mechanism operates for HSV-1 then antibody-based detection of UL36p on nuclear capsids would yield variable results, depending upon whether the relevant epitope is present within the capsid-bound UL36p fragment.

## 3. Capsid Transport and Envelopment in the Cytoplasm 

### 3.1. Reorganization of Microtubules in the HSV-1 Infected Cell

Whether inner tegument is attached to capsids within the nucleus, or soon after emergence into the cytoplasm, egressing HSV-1 capsids are similar in structure and composition to the virions that enter a cell upon initial infection. Both are non-enveloped DNA-packaged capsids with associated inner (and perhaps also some outer) tegument proteins. Nevertheless, infecting capsids must recruit dynein for MT-mediated retrograde transport to the nucleus, while those undergoing egress require kinesin motor attachment for anterograde transport to the cell periphery. The state of the host-cell MT network may be one cue that helps HSV-1 capsids choose the correct transport destination. During HSV-1 infection of epithelia and fibroblasts the microtubule-organizing center (MTOC) becomes disrupted, and MT growth originates from multiple new nucleating centers throughout the cytoplasm [63,75,116]. This reorganization may well facilitate assembly and egress of progeny virions since the new nucleating centers include the trans-Golgi network (TGN) [116], an organelle proposed to be the site of HSV-1 capsid envelopment (Section 3.3). Changes in MT nucleation are directed at least in part by the inner tegument serine/threonine kinase US3p (Table 1, Section 2 and Section 4.5) [28,63,65]. US3p inactivates the host cell glycogen synthase kinase-3β (GSK3β), de-repressing cytoplasmic linker-associated proteins (CLASPs) that regulate MT stabilization and growth, including from the TGN [63]. MTs are also stabilized by hyperacetylation and bundling induced by the outer tegument MT-binding protein UL49p (VP22) (Table 1, Section 3.6) [75,76,77]. MT acetylation may also have consequences for motor selection by egressing virions; in neurons acetylation at one of the most common and conserved acetylation sites, the lysine-40 residue of α-tubulin [117] generates MTs that are a favored substrate for the kinesin-1 motor KIF5 [118]. Thus, post-translational stabilization of subsets of MTs, assembly of novel infected cell-specific MT nucleating centers, and control of motor choice could direct traffic of non-enveloped HSV-1 capsids away from the nucleus, toward sites of cytoplasmic envelopment and subsequently to the cell surface (Section 4 and Section 5). Perhaps the most dramatic example of MT reorganization during HSV-1 infection is the US3p-induced generation of MT-containing tunneling nanotubes (TNTs) [119,120], extended processes that reach from infected to uninfected cells and that may provide dedicated highways for HSV-1 spread. These are discussed in greater detail in Section 4.5. 

### 3.2. Trafficking of Non-Enveloped HSV-1 Capsids along Microtubules

**Figure 2:**
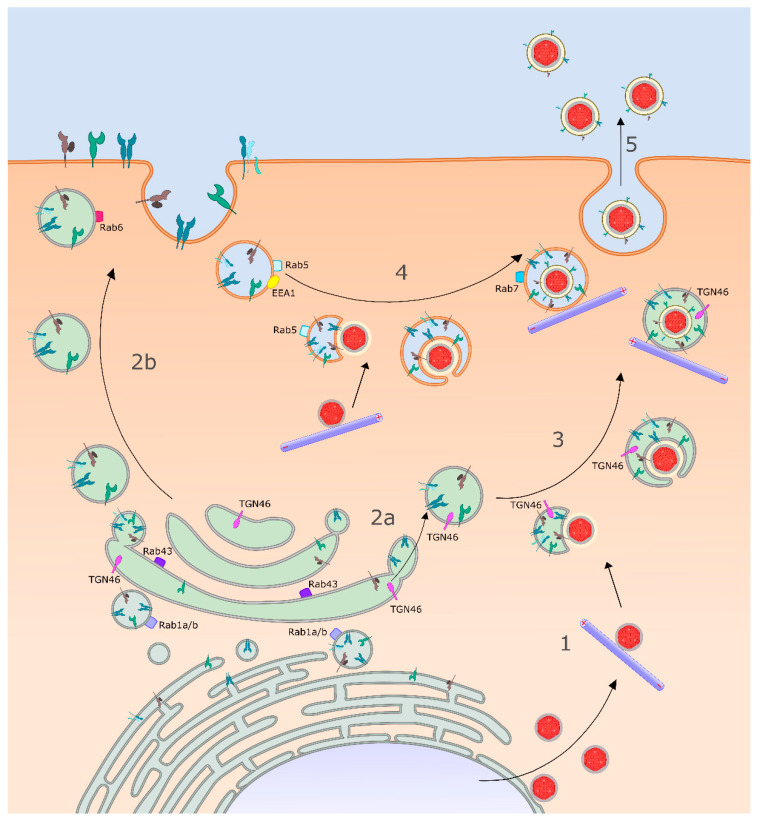
HSV-1 cytoplasmic envelopment and trafficking. (1) HSV-1 capsids (red hexagons) emerge from the nucleus and inner tegument (gray layer) recruits kinesin for traffic to the plus ends of MTs (purple cylinders with + and - ends indicated) and to sites of cytoplasmic envelopment. Meanwhile, viral envelope proteins are processed via the conventional secretory pathway and traffic from the endoplasmic reticulum (ER) to the TGN (2a) and/or plasma membrane (2b). Rab1a/b (light purple squares) facilitate envelope protein export from the ER, while Rab43 (purple squares) supports transport through the Golgi apparatus and Rab6 (red square) is important for delivery of envelope proteins to the cell surface. (3) In one model HSV-1 capsids acquire their envelopes by attaching to, and budding into, the TGN or TGN-derived vesicles that contain the integral membrane protein TGN46 (violet lollipops). (4) Alternatively, cell-surface viral envelope proteins are endocytosed in a Rab5 (pale blue squares)-dependent manner into tubular–vesicular structures that provide the envelope. However, these tubular-vesicular structures lack the marker EEA1 (yellow oval) and thus are distinct from early endosomes. In other studies, egressing HSV-1 capsids have been observed to colocalize with early and late endosomes (Rab5- and Rab7-labeled, respectively). In all cases capsid envelopment is coupled to completion of the outer tegument (yellow layer around capsid) and incorporation of envelope proteins into the mature virion. (5) OEVs utilize MTs to deliver mature enveloped virions to the cell surface. See text for details.

HSV-1 capsids utilize MT-directed anterograde transport and kinesin motors to travel through the cytoplasm to the organelles at which they acquire their mature envelope (Figure 2). Biochemical studies revealed that purified non-enveloped HSV-1 capsids can bind kinesin-1 and kinesin-2 in vitro, but only if the inner tegument proteins UL36p and UL37p are present and not obscured by outer tegument [53]. Consistent with these data, UL36p-null HSV-1 capsids could not traffic along MTs in an in vitro system [57,121], and deletion of the UL36 or UL37 genes disrupted MT-directed anterograde HSV-1 capsid traffic in epithelial cells and mouse dorsal root ganglia (DRGs). These UL36p-null and UL37p-null capsids demonstrated random, undirected diffusion in the cytoplasm [56,122]. Taken together, these data suggest that after UL36p binds UL37p to the capsid, UL37p in turn recruits kinesin motors, or UL36p and UL37p cooperate to create a kinesin binding site [56,122,123]. Interestingly, mutation of two tryptophan motifs conserved in all available HSV-1 and HSV-2 UL36p protein sequences (W^1766^D^1767^ and W^1862^E^1863^ in HSV-1 strain 17^+^) inhibited envelopment and led to accumulation of non-enveloped capsids in the vicinity of the MTOC, though the mutant UL36p protein attached to capsids and recruited UL37p normally [54]. These WD/WE “tryptophan-acidic” motifs resemble the bipartite signal that cargo proteins use to associate with the tetratricopeptide repeat (TPR) motif of kinesin light chains (KLCs) [124,125,126], and thus could be part of the mechanism by which UL36p contributes to kinesin recruitment [53]. If so, mutation of the UL36p WD/WE motifs abolishes capsid envelopment because of failure to recruit kinesin, and thus inefficient MT-directed anterograde traffic to the envelopment organelle [54]. Alternatively, or in addition, the conserved tryptophan residues might be essential for UL36p conformational changes or intermolecular associations that play more direct roles in envelopment [54] (Section 3.6). 

In addition to being important for kinesin recruitment, UL37p has been shown to associate with dystonin/bullous pemphigoid antigen 1 (BPAG1) [127] in a yeast two-hybrid assay [128]. Dystonin is a large protein with multiple isoforms that interacts with the actin cytoskeleton and with clathrin, and that plays roles in MT stability and Golgi organization [127,129]. It has also been proposed to connect membrane-imbedded receptors on organellar cargo with the dynein/dynactin complex [130,131,132]. Given the role of dynein/dynactin in retrograde movement along MTs [131], it is striking that dystonin depletion inhibited *anterograde* trafficking of HSV-1 capsids through the cytoplasm during egress in human fetal foreskin fibroblast 2 (HFFF2) cells [128]. The role of dystonin in HSV-1 egress, and its relationship with UL37p in anterograde transport, remain to be established [5,128]. 

### 3.3. Identity of the HSV-1 Cytoplasmic Envelopment Organelle

HSV-1 capsids traffic to, and dock with, the cytoplasmic surface of organelles that provide the lipid bilayer of the envelope and the full complement of 17 virally encoded envelope proteins [11]. Of these, 12 are attached to N-linked sugars and are named by a letter and the prefix g (glycosylated): gB, gC, gD, gE, gG, gH, gI, gJ, gK, gL, gM, and gN [11,133,134]. Glycoproteins gD, gB, and the gH/gL obligate heterodimer comprise a “core” fusion apparatus that is sufficient for cell–cell fusion in the absence of other viral gene products [81] and essential for HSV-1 entry into target cells in tissue culture and in animal models [133,134,135]. The remaining 5 envelope proteins are not thought to be glycosylated: UL20p, UL43p, UL45p, UL56p, and US9p [11,133,134]. HSV-1-encoded membrane-associated proteins that are relevant to this review are summarized in Table 1. The identity of the organelle utilized for HSV-1 envelopment, and mechanisms that ensure delivery of envelope proteins to it, are discussed below.

HSV-1 capsids have been observed enveloping at structures morphologically resembling the TGN and that stain for TGN marker antigens and lipids (Figure 2) [136,137,138]. Furthermore, envelopment at the TGN or the Golgi apparatus would be consistent with the sphingomyelin and phosphatidylserine content of the envelope of mature HSV-1 particles [139]. We demonstrated that during a single synchronized wave of HSV-1 trafficking from the nucleus through the cytoplasm [140], capsids bypassed the cis, medial, and trans compartments of the Golgi apparatus [141] but accumulated in a subcellular density gradient fraction enriched in the TGN marker TGN46 and early and late endosomal markers Rab5 and Rab7 [141,142]. In other studies UL37p, which clearly plays important roles in capsid transport (Section 3.2), organelle docking (Section 3.5), and envelopment (Section 3.6) [56,62,122] was found to localize to the Golgi or TGN [143]. UL37p required UL36p for Golgi/TGN-association, but not the presence of HSV-1 capsids [143]. These data suggest that UL36p/UL37p could bridge the capsid shell and the lipid bilayer of the Golgi/TGN and thus exist both in the inner tegument and as a peripheral membrane complex anchored to the cytoplasmic face of the Golgi/TGN. In this regard, while wild-type HSV-1 capsids were reported to colocalize with the TGN and to envelope there [123], UL37p-null capsids were instead dispersed throughout the cytoplasm [123]. However it is unclear whether this UL37p-null phenotype was due to a capsid-TGN docking/envelopment defect or a failure in kinesin recruitment and MT-directed traffic to the envelopment site [56,122] (Section 3.2). 

Studies of the intracellular sorting of HSV-1 particles have also identified the TGN as a key player. Turcotte and colleagues [144] used the classic cell biology technique of a reduced temperature “20 °C block” to arrest export out of the TGN [145,146] in baby hamster kidney (BHK) and human 143B osteosarcoma tumor cells [144]. Under these conditions most transport-arrested HSV-1 capsids colocalized with the TGN marker TGN46 and the Golgi/TGN lipid marker NBD ceramide, but not with markers of the lysosomes, ER, or other Golgi compartments. However, some capsids did colocalize with the early endosomal marker EEA1 [136,144]. Trafficking of HSV-1 from the TGN in 143B cells appeared to be within carrier tubules or vesicles similar to those utilized for sorting of normal cellular cargo [147,148,149], and like conventional TGN cargo the “pinching-off” or scission of these carriers required the activity of cellular protein kinase D (PKD) [149]. 

The TGN has also been proposed to be the location from which enveloped HSV-1 particles are sorted to lateral (and away from apical) surfaces of polarized epithelial cells by a gE/gI-dependent pathway during cell–cell spread [22,87,150] (Section 4.1 and Section 4.2). Interestingly, the cellular TGN residents TGN46 and carboxypeptidase D (CPD) also redistribute to lateral cell–cell junctions during HSV-1 infection [151]. This may reflect a general reorganization of the exocytic pathway to favor delivery of cargo virions to the cell surface, though relocalization of TGN46 and CPD did not require the assembly of enveloped HSV-1 particles [151]. The redistribution of these proteins from the TGN however demonstrates that care must sometimes be taken when using marker antigens to identify organelles in HSV-1 infected cells.

An alternative model for the source of the HSV-1 envelope, based in part upon studies in HFFF-2 fibroblasts, is that capsids become wrapped by envelope protein-containing endocytic tubules derived from the plasma membrane [152] (Figure 2). In this scenario viral envelope proteins traffic via the conventional secretory pathway from the ER to the Golgi apparatus, then from the TGN to the cell surface in a Rab6-dependent manner [142,153]. Once at the plasma membrane the envelope proteins are retrieved by dynamin and Rab5-regulated endocytosis into tubular structures that are utilized for envelopment [142,152]. Albecka and colleagues, working with COS7 monkey kidney fibroblasts, HFF-Tert telomerase-immortalized human foreskin fibroblasts, and HaCaT human keratinocytes, also concluded that envelope glycoproteins were delivered to HSV-1 capsid envelopment organelles following dynamin-dependent endocytosis from the cell surface [154]. The tubular endocytic organelles utilized for envelopment in these studies appeared to be distinct from early and late endosomes, since they lacked the markers EEA1 and LAMP2 [152]. Accumulation of envelope proteins in these tubular compartments was clearly important for virion assembly; depletion of Rab6 [142] reduced trafficking of envelope proteins to the cell surface, prevented their internalization to the endocytic compartment, and inhibited capsid envelopment, reducing HSV-1 yields by 99% [153]. Similarly, blocking viral glycoprotein endocytosis from the cell surface by depletion of Rab5 generated endocytic tubules unable to support envelopment, and a 95% reduction in HSV-1 titers [152]. In separate studies knockdown of Rab1a/b and Rab43 also diminished viral titer, in this case apparently due to failure of envelope proteins to leave the ER or because of effects upon the integrity and/or identity of post-Golgi assembly compartments (respectively) [155]. 

Strikingly different conclusions were reached concerning the importance of endocytosis in delivery of membrane proteins for PRV envelopment [156]. Although PRV US9p and the heterodimer gE/gI (Section 4.1) were clearly endocytosed from the cell surface they were nevertheless sorted to distinct compartments, the TGN and endosomal-recycling pathway respectively [157,158,159]. Furthermore, blocking endocytosis of PRV US9p and gE/gI by ablation of endocytic motifs in the cytoplasmic tails of gE and US9p, and even removal of the entire gE cytoplasmic tail, had no effect upon incorporation of the mutant proteins into the PRV envelope [157,159,160,161,162]. Indeed, internalization of PRV gB and gE, and the cellular transferrin receptor, were actually inhibited as early as 6 h post-infection, suggesting a global shutdown of receptor-mediated endocytosis before the majority of infectious PRV virions are constructed [156,158]. It is unlikely that early waves of endocytosis generate a reservoir of envelope proteins that are subsequently utilized for envelopment since gE molecules internalized at 4 h post-infection were not present in virions assembled at later times [158]. It is unclear why HSV-1 and PRV, which exhibit so many similar properties concerning replication and spread in epithelial cells and sensory neurons [5,7], should be so different in their use of the endocytic pathway for envelope protein targeting and capsid envelopment. 

An appealing feature of the endocytosis model for HSV-1 envelopment is that it provides a role for the functional endocytic motifs known to reside in the cytoplasmic tails of several envelope proteins including gB (Section 3.4), gE/gI, and US9p (Section 4.1) [78,82,84,87,89,163]. Nevertheless, for gE/gI these sorting signals were reported to target the heterodimeric complex to the TGN [87,151,163]. Similarly, internalization motifs within the cytoplasmic domain of gB are thought to target gB to the TGN [78,82] or to multivesicular bodies [79] for HSV-1 capsid envelopment. One way of explaining these results is to postulate that TGN provides a “safety net” to capture envelope proteins that reached the endocytic envelopment organelle but then trafficked beyond it. In this model, envelope proteins that escape the envelopment site would be captured by the TGN and returned to the cell surface for another round of endocytosis. This suggestion is speculative, but mutation of the endocytic motifs in the gB cytoplasmic tail do affect the distribution of gB between the cell surface, recycling endosomes and the TGN [78,82]. It is also important to bear in mind that several envelope proteins must be sorted to additional subcellular destinations to serve roles unrelated to envelopment. For example, in polarized epithelial cells one reason that gE/gI and gB may localize to the TGN is that this is the site from which they are sorted to cell–cell junctions to promote lateral sorting of HSV-1 particles and cell–cell spread [22,150,151,164] (Section 4.2). 

Cycling of envelope proteins between the cell surface, endosomal compartments, and TGN might provide another means of reconciling conflicting data, suggesting the TGN or endocytic tubules as sites of envelopment. As described above, even in studies concluding that envelopment occurs at the TGN, some capsids were found in association with the early endosomal marker EEA1 [136,144]. This result was interpreted to suggest that viral envelope proteins, recycling between the TGN and endosomes [144], might make a subpopulation of endosomes resemble the surface of TGN, leading to capsid association with those organelles. We similarly observed HSV-1 capsids associating with the TGN, but also to a lesser extent with Rab5- and Rab7-labeled endosomes, in Vero cell extracts [57,58]. Importantly, the capsid envelopment experiments we have reviewed above used a variety of different cell lines. Perhaps envelopment at endocytic tubules occurs in those cell types, or under those growth conditions, where envelope proteins exit the endocytic compartment relatively slowly. Cell lines or conditions favoring rapid delivery of endocytic cargo to the TGN might shift capsid envelopment to that location. 

### 3.4. Delivery of Envelope Proteins to the Site of Envelopment: Roles for gK/UL20p and gM

Multiple HSV-1 encoded membrane proteins must be delivered to the envelopment organelle to support assembly (Section 3.6) and to ensure that the mature virion is fully infectious [11,133,134]. At least some envelope proteins, for example gD and the heterodimer gH/gL (Table 1), use virally encoded mechanisms to ensure efficient sorting to the correct bilayer. Both gD and gH/gL lack recognizable endocytic motifs and accumulate on the cell surface when expressed in the absence of other viral proteins, but become sorted to intracellular locations (presumably including the site of envelopment) during HSV-1 infection [96]. An important component of the sorting apparatus is gK/UL20p (Table 1), a complex of two HSV-1-encoded multi membrane-spanning domain envelope proteins [91,92]. gK/UL20p is capable of endocytosis in the absence of other HSV-1-encoded proteins, and its expression inhibits the cell–cell fusion that normally occurs when the “core” HSV-1 fusion-machinery glycoproteins gH/gL, gB, and gD accumulate on the cell surface [93]. This suggests gK/UL20p retrieves these envelope proteins from the plasma membrane, preventing their participation in cell–cell fusion and perhaps delivering them to the intracellular site of envelopment. This would be consistent with HSV-1 envelopment at endosome-derived tubules as described above (Section 3.3), although endocytosed gK/UL20p appears to be targeted to the TGN rather than to tubular endosomal-like compartments [92]. A similar envelope protein sorting function has been ascribed to the HSV-1 multi-membrane-spanning protein gM, known to exist (at least in part) in a TGN-localized heterodimeric complex with the membrane protein gN (Table 1) [94,96]. Analysis of HSV-1 mutants lacking gK, UL20p, and gM, singly and in combination, revealed that both gK/UL20p and gM (or gM/gN) can mediate the internalization of gD and gH/gL from the cell surface [90]. Moreover, gK/UL20p and gM were both required for efficient gH/gL incorporation into HSV-1 particles [90,95]. However, gK/UL20p and gM do not always perform similar tasks. Although gM was sufficient to internalize normal levels of gD from the cell surface, this gD was not efficiently incorporated into virions unless gK/UL20p was present [90,96]. This suggests that gK/UL20p, but not gM, mediates the entry of gD into a particular subset of endosomes able to deliver gD to the envelopment membrane, or that gK/UL20p ensures post-endocytic sorting essential for correct targeting of gD [90]. Coimmunoprecipitation of gK and UL20p with gB [165] raises the possibility that gK/UL20p controls the trafficking of this envelope protein too, even though it has its own endocytic motifs [78,82] (Section 3.3). Alternatively, gK/UL20p may interact with gB to control its fusogenic activity during HSV-1 entry or cell–cell fusion [165]. Interestingly, UL20p coimmunoprecipitates with gM [166], implying communication between the gK/UL20p and gM protein trafficking pathways. It is also possible that gK/UL20p and gM (or gM/gN) are actually components of a single-sorting apparatus whose subunits share distinct or overlapping responsibilities for the targeting of particular HSV-1 envelope proteins to the envelopment bilayer.

### 3.5. Capsid Docking to the Surface of the Envelopment Organelle

In addition to its role in recruitment of kinesins for cytoplasmic trafficking of non-enveloped HSV-1 capsids (Section 3.2), the amino-terminal portion of UL37p (UL37Np) bears structural similarity to cellular MTCs (Section 2), which help tether transport vesicles to their destination membranes prior to fusion [42,43]. The four MTC subunits with the highest structural similarity to UL37Np are components of the Dsl1 complex (Tip20 and Dsl1) and the exocyst complex (Sec6 and Exo70), which coordinate tethering of Golgi apparatus-derived vesicles to the ER and plasma membrane, respectively [42]. Thus, UL37p might help dock capsids to the surface of their envelopment organelle [123], possibly via binding to the envelope protein heterodimer gK/UL20p [43,60,61] (Table 1, Section 3.4). A role for UL37p in docking is consistent with earlier findings that loss of UL37p disrupted capsid association with, and envelopment by the TGN [62,123] and the observation that the fidelity of capsid targeting to the TGN was impaired in the absence of UL36p (and thus also UL37p) [58]. We observed that, although UL36p/UL37p-null HSV-1 capsids efficiently associated with organellar membranes [57], a subpopulation of capsids shifted from the TGN to Rab5- and Rab7-labeled early and late endosomes [57,58]. In addition to being mislocalized [57,58] some UL36p-null HSV-1 capsids also aggregated on the surfaces of membranes [58] as well as in the cytoplasm [58] as originally established [55]. Nevertheless some individual, non-aggregated UL36p-null HSV-1 capsids could be observed attached to the surfaces of organelles, apparently connected by fine fibers [59]. We have speculated [58,59] that the docking apparatus remaining on UL36p/UL37p-null capsids might be the Golgi-anchored UL11p/UL16p/UL21p complex characterized by the Wills laboratory (Table 1) [47,49,50,167,168]. UL11p/UL16p/UL21p is an attractive candidate for this task since it can connect to organellar surfaces via attachment to the cytoplasmic tail of gE or through its UL11p lipid anchors [48,51,169] and because UL16p is the rare example of an “outer” tegument protein able to bind capsids independently of UL36p and UL37p [10,48,50,51]. An important role for UL16p in capsid-membrane interaction is consistent with the striking phenotype resulting from its absence; not only defective cytoplasmic envelopment but also the appearance of structures resembling individual envelopes attempting to enclose multiple capsids [170]. The existence of an alternative, and perhaps redundant, capsid/membrane docking apparatus is also implied by the finding that the MTC-like UL37Np domain is dispensable for HSV-1 replication. Deletion of the amino-terminal 480 amino acids of UL37p resulted in only a five-fold decrease of titer in epithelial cell lines [44]. Interestingly, smaller deletions from the amino-terminus of UL37p were lethal and caused an envelopment defect, suggesting these truncation mutants may be defective in docking or envelopment but still able to interfere with it [44]. In contrast, truncations in the carboxy-terminal half of UL37p were more likely to result in severe replication and envelopment problems [44] phenocopying a complete UL37 null virus [62]. Carboxy-terminal deletions could be affecting the interaction of UL37p with UL36p [39], dystonin/BPAG1 [127,128,171], or gK/UL20p [43,60,61] (Table 1); hence, the reasons for the envelopment defect remain to be determined. 

### 3.6. Capsid Envelopment and the Roles of HSV-1 Encoded Envelope and Tegument Proteins

After docking with the envelopment bilayer capsids bud into the organellar lumen in concert with recruitment of envelope and outer tegument proteins (Figure 2). Generation of envelope curvature, and scission and sealing of the envelope neck, require the coordinated activity of multiple viral envelope and tegument proteins and the cellular endosomal sorting complex required for transport (ESCRT) apparatus [11,12,13,22]. 

A number of HSV-1 encoded envelope and tegument proteins are known to be important for capsid envelopment [11,12,13,67] (Table 1). These molecules may play roles in outer tegument assembly (Figure 1, Section 2), collection of envelope proteins into the budding site (Section 3.4), capsid-membrane docking (Section 3.5), directly participate in curving the lipid bilayer around the capsid or recruit cellular ESCRT complexes (Section 3.7) to support envelope wrapping and scission [11,12]. In most cases deletion of individual tegument and envelope protein genes has little effect on envelopment, with serious defects arising only when two or more are removed simultaneously. For example, loss of genes encoding gB or gD resulted in a 2- to 5-fold increase in numbers of non-enveloped or partially enveloped capsids in the cytoplasm, but a 15-fold increase when gD and gB were deleted at the same time [80]. Similarly, loss of gD and gE, or gD and the gE/gI heterodimer (Section 4.1), caused a 20- to 40-fold increase in the numbers of non-enveloped cytoplasmic capsids, while individual loss of gD or gE had marginal effects [83]. Removal of gE combined with loss of the small integral membrane protein US9p (see Section 4.1 for more details concerning this molecule) led to a 10- to 12-fold increase in numbers of non-enveloped or partially enveloped cytoplasmic capsids, but only in cells of neuronal origin, showing that envelopment may have differential requirements across cell types [84]. In general, even multiple deletions result in a modest (2 log or lower) reduction in numbers of enveloped particles or in viral titer, suggesting broad redundancy in the envelopment roles performed by envelope and tegument proteins [11,13,22,23]. 

Since simultaneous deletion of several envelope protein genes can impact envelopment [84,172,173] it might be expected that failure to deliver multiple envelope proteins to the site of envelopment would have a similar effect. As already discussed (Section 3.4) the heterodimer gK/UL20p [60,61,165,174,175,176] and gM (or gM/gN) [172,176] play important and partially redundant roles in targeting at least some envelope proteins to the site of envelope assembly, and indeed removal or mutation of gK or UL20p results in HSV-1 titer and capsid envelopment defects [90,174,176,177,178]. The envelopment defect was found to become more severe when gK or UL20p were deleted at the same time as gM [90], though loss of gM alone caused only modest reductions in titer and a slight increase in numbers of non-enveloped cytoplasmic capsids [90,179]. The mild envelopment defect resulting from loss of gM was also exacerbated by deleting UL11 [46], which encodes the small, myristoylated, palmitoylated tegument protein UL11p (Table 1) found as part of the TGN-anchored UL11p/UL16p/UL21p complex (Figure 1, Table 1) described in Section 3.5 [47,49,50,167,168]. This finding is consistent with the notion that the lipid-anchored peripheral membrane protein UL11p reaches the capsid envelopment bilayer via a gM-independent route, and provides assembly functions distinct from those of gM-delivered integral-membrane envelope proteins. In PRV the simultaneous loss of gM and gE/gI (but not loss of gE/gI alone) resulted in a dramatic failure of capsid envelopment, with accumulation of non-enveloped cytoplasmic capsids in association with aggregated electron-dense material resembling tegument [173]. This suggests that PRV gE/gI provides envelopment functions that are dispensable when gM is present, perhaps because gM delivers other envelope proteins that perform similar tasks. However, simultaneous deletion of gE/gI and gM in HSV-1 had marginal effects upon replication and envelopment [172]. Hence, for these two alphaherpesviruses, envelope proteins might utilize different mechanisms for delivery to the site of envelopment (HSV-1 gM might not be required for delivery of envelope proteins that complement the lack of gE/gI) or the “distribution of effort” between envelope proteins driving HSV-1 and PRV envelopment differs. Further illustrating the latter possibility, although simultaneous loss of gE/gI and US9p impairs HSV-1 capsid envelopment in cultured neurons (see above), identical deletions have no measurable effect upon PRV envelopment in the same host cell background [180]. A final point to consider is that, although it is clear that gK/UL20p and gM (or gM/gN) modulate the trafficking of envelope proteins to the envelopment site [90,95,96], gK/UL20p and gM may also directly participate in envelopment (a role for gK in UL37p-mediated HSV-1 capsid-membrane docking has already been discussed in Section 3.5).

Eleven tegument proteins: UL7p, UL11p, UL16p, UL21p, UL36p, UL37p, UL46p (VP11/12), UL47p (VP13/14), UL48p (VP16), UL49p (VP22), and UL51p [11,12,13,67] have been implicated in HSV-1 cytoplasmic envelopment (Table 1), usually on the basis of the phenotype resulting from deletion of the corresponding genes (reviewed in detail in [11]). The inner tegument components UL36p and UL37p have already been discussed in detail, and have long been known to be essential for envelopment [55,62] likely as a result of their multiple roles including assembly of outer tegument (Section 2), MT-mediated capsid transport (Section 3.2), capsid-membrane docking (Section 3.5), and possibly also coupling HSV-1 envelopment to the cellular ESCRT apparatus (see below, Section 3.7). 

UL46p (VP11/12), UL47p (VP13/14), UL48p (VP16), and UL49p (VP22) (Table 1) are among the most abundant tegument components in the mature virion. They form multiple interactions with other tegument proteins, and UL47p (VP13/14) also binds to UL17p [70], a component of the UL36p-anchoring complex at the capsid vertices (Figure 1, Section 2). VP22 also interacts with the tails of both gD and gE, connecting the outer tegument to the inner envelope [11,74,181] (Table 1, Figure 1). Nevertheless, of these four proteins only UL48p (VP16) is considered essential in tissue culture [11,71,72], perhaps because of its pivotal role in connecting inner tegument UL36p to multiple outer tegument and envelope proteins [11,35,36,73] (Section 2, Figure 1). 

We have already described the UL11p/UL16p/UL21p complex (see above, and Section 3.5) that binds HSV-1 capsids and also attaches to the cytoplasmic tail of gE [47,49,50,51,167,168]. This Golgi-anchored complex may help dock HSV-1 capsids to the envelopment membrane (Section 3.5) and loss of its UL11p subunit exacerbates the envelopment defect that results from loss of gM [46] (see above). Similarly, loss of UL16p results in a defect in envelopment in which multiple capsids appear to be partially enclosed by a single envelope [170] (Section 3.5).

Similar to UL11p, UL16p, and UL21p, the UL51p and UL7p proteins form a lipid-anchored membrane-associated complex (Figure 1), though UL51p/UL7p exhibits only partial overlap with Golgi markers [66,182,183]. Deletion of UL51 resulted in a ~100-fold decrease in HSV-1 titer but the terminal phenotype appeared to be accumulation of enveloped HSV-1 particles in the perinuclear space, suggesting a de-envelopment defect in nuclear egress [184]. In contrast, other studies concluded that loss of UL7p/UL51p led to an HSV-1 cytoplasmic envelopment defect [67], as reported for a UL51-null mutant of PRV [185]. Recent structural analyses indicates that UL7p and UL51p may recruit the cellular ESCRT apparatus to support envelopment and scission [67,69], discussed in greater detail below (Section 3.7). 

Taken together, the data above suggest that virally encoded envelope and tegument proteins cooperate in a rather flexible and redundant manner to drive envelopment, and that the distribution of effort across these molecules varies between different alphaherpesviruses and host cell backgrounds. How might viral envelope and tegument proteins, with little or no primary sequence in common, nevertheless share redundant envelopment functions? One possibility is that there are multiple independent parallel mechanisms to reshape the lipid bilayer into the envelope. Loss of, or reduced function in, one pathway could then be compensated by enhancing the activity of another. Alternatively, Metrick and colleagues [45] recently proposed that all inner and outer HSV-1 tegument proteins contain intrinsically disordered regions (IDRs), of variable length, which enable them to undergo phase separation, forming a spherical biomolecular condensate that helps drive envelopment [186]. If the tegument exists (at least in part) as a biomolecular condensate, it would be extremely tolerant to loss or change in expression level of many of its constituents, and potentially quite variable in its final composition, as multiple tegument or even host proteins could contribute to condensate formation [45,187,188]. Of possible relevance to this model, we found that the tegument protein UL48p (VP16) (Table 1) binds in vitro to a peptide with the sequence of the gH cytoplasmic tail, but only at 37 °C when that peptide was in a disordered state [35,36]. As the temperature was reduced to 25 °C and below the peptide gradually became more structured due to formation of a tight turn at a central proline residue, and simultaneously lost the ability to bind UL48p (VP16). Replacing the central proline with an alanine resulted in a peptide which was unstructured at all temperatures tested, and able to bind UL48p (VP16) even at 4 °C [35]. It is therefore possible that disordered regions support both tegument assembly and the association of tegument with membrane protein tails at the inner surface of the envelope.

### 3.7. The Cellular ESCRT Apparatus in Envelopment and Scission

The final step in envelopment is scission, pinching-off, and sealing the envelope to form a mature infectious virion in the lumen of the surrounding organelle. Alphaherpesviruses, in common with many other families of enveloped viruses, utilize the cellular ESCRT apparatus for scission and possibly earlier steps in envelope assembly [11,12,189,190,191]. Details of the ESCRT machinery and its roles in envelopment of herpes and other viruses have recently been reviewed [12,190,192]. In brief, the ESCRT apparatus directs assembly of ESCRT-III, a spring-like polymer of charged multivesicular body protein (CHMP) subunits, on the cytoplasmic surface of an organelle. ESCRT-III then drives deformation of the flat lipid bilayer into vesicles or tubules “away” from the cytosol, into the organellar lumen or outward from the plasma membrane [189] though it can also form membrane tubules with the opposite orientation [193]. ESCRT-III then constricts, resulting in scission or pinching-off at the vesicle or tubule neck, a process coupled to ESCRT-III disassembly by the hexameric AAA ATPase vacuolar protein sorting 4 (Vps4) [189,192,193,194,195]. Polymerization of CHMPs to form ESCRT-III fibers is catalyzed by the complexes ESCRT-I, ESCRT-II [192,194], and proteins containing the Bro1 domain, a ~390 amino acid long banana-shaped module found in several cellular proteins including Alg-2 interacting protein X (ALIX) [189,194], His domain-containing protein tyrosine phosphatase (HD-PTP) and Bro1 domain/CAAX motif containing-protein (BROX) [196]. Enveloped viruses have evolved various means to recruit ESCRT complexes and Bro1-domain proteins to catalyze ESCRT-III polymerization at the necks of their envelopes [12,190], though the mechanism of ESCRT recruitment by herpesviruses has been elusive [12,191,197,198]. Two common viral strategies to control ESCRT-III assembly are recruitment of ALIX and/or the ESCRT-I complex (in the latter case via its subunit tumor susceptibility gene 101 [TSG101]) [12,190]. However, TSG101 and ALIX are dispensable for HSV-1 envelopment, even when depleted simultaneously [198]. Similarly, knockdown of the critical EAP20/VPS25 subunit of ESCRT-II, and of the remaining known ESCRT-associated Bro1 domain proteins HD-PTP and BROX, had no effect upon HSV-1 replication [199]. 

Given these data, it is reasonable to hypothesize that ESCRT-III might be directly recruited by proteins resident in the HSV-1 tegument or envelope, particularly as deletion of the foundational inner tegument components UL36p or UL37p [11,13,40,55,62] and mutations within them [44,54] all abolish cytoplasmic envelopment. We found that loss of UL36p reduced the efficiency with which membrane-associated HSV-1 capsids were able to engage with the ESCRT apparatus by approximately 70%, suggesting that UL36p plays an important role in recruitment of capsids to the site of ESCRT-III/Vps4 deposition [59]. Indeed, UL36p has been reported to interact with the ESCRT-I subunit TSG101, and to modulate its ubiquitination [200]. Nevertheless, as mentioned above, TSG101 is dispensable for HSV-1 envelopment [198] and UL36p is required for recruitment of multiple outer tegument proteins into the enveloping particle [25,33,34,41,43,201] (Section 2, Figure 1), so its role in capsid/ESCRT-III/Vps4 assembly is likely to be indirect [59]. 

A recent study has demonstrated that the tegument protein UL51p [66,67] (Table 1) has striking structural similarity to the ESCRT-III subunit CHMP4B, and can self-associate to form long ESCRT-III-like polymers in vitro [69]. This raises the exciting possibility that UL51p catalyzes polymerization of cellular CHMP subunits, or may even substitute for them, to control formation of an ESCRT-III or ESCRT-III-like filament. One copy of the tegument protein UL7p assembles with two copies of UL51p to form a UL7p/(UL51p)_2_ TGN-associated heterotrimeric complex, and bound UL7p inhibits UL51p self-polymerization, suggesting a mechanism by which the positioning and timing of ESCRT-III assembly might be regulated [67,69]. 

A feature of tissue culture replication common to all alphaherpesviruses examined is the production of light (L)-particles, similar in size to mature virions (though more heterogeneous) and consisting of lipid envelope-like structures with membrane-imbedded envelope proteins and tegument, but lacking capsids [11,202,203,204,205,206]. These non-infectious particles are thought to arise when capsids fail to engage with the cytoplasmic envelopment machinery, but where envelopment nevertheless still occurs [11], presumably driven by tegument, envelope proteins and the ESCRT-apparatus [11,12]. If the envelopes of L-particles and mature HSV-1 virions are indeed assembled using similar molecular machinery, then the composition of L-particles may help identify proteins that are important for delivering structural components to the envelope, bending it and driving scission of the lipid bilayer rather than, for example, attaching it to HSV-1 capsids. Considering the data discussed above it is therefore interesting that, relative to mature infectious HSV-1 virions, HSV-1 L-particles are slightly enriched for UL51p, gK/UL20p, and most envelope glycoproteins, but depleted for UL36p and UL37p [206]. 

### 3.8. MT-Directed Transport of Enveloping Capsids Is Arrested until Envelopment Is Complete

The envelopment of HSV-1 capsids to generate OEVs (Figure 1 and Figure 2) is an important transition in the trafficking problem for this virus. Non-enveloped capsids are relatively small, ~125 nm diameter proteinaceous particles. In contrast, OEVs are at least ~200 nm in diameter [59,191,207] or larger if multiple enveloped virions are accommodated within the carrier organelle. OEVs will require greater force to move them along MTs than is required for capsids, and their bounding lipid membrane creates a far more flexible surface for motor attachment than the rigid capsid shell. Members of the kinesin family differ considerably in their force generation and their propensity to operate in teams to move large or small cargo [208,209,210], so it is quite likely that different anterograde motors become utilized by non-enveloped capsids and OEVs during transport. 

HSV-1 capsids can be trapped in a partially enveloped state by expression of Vps4-EQ, an ATPase-deficient allele of Vps4 (Section 3.7) that has a dominant negative effect on Vps4/ESCRT-III-driven scission [189,193,194,211]. In the presence of Vps4-EQ, capsids are only partly wrapped by the envelopment organelle, leaving the capsid shell exposed [59,191,197]. Using a GFP-tagged Vps4-EQ we were able to visualize these envelopment intermediates in cell extracts by fluorescence microscopy, and found that they were able to bind to MTs in an in vitro system [197]. However, the envelopment intermediates were incapable of trafficking along the MTs in vitro, even though MT-directed trafficking of capsids and mature sealed OEVs was normal [197]. Although this phenomenon remains to be demonstrated in vivo, we have hypothesized that these data suggest the existence of an assembly checkpoint. Once HSV-1 capsids have entered the envelopment pathway any further MT-dependent trafficking is prevented until OEVs have been correctly assembled and the cargo of infectious virions is ready for export [197]. Such a checkpoint might also provide a convenient juncture for the removal of capsid-associated kinesins, and the loading of kinesins more suited for OEV transport onto the bounding membrane of the envelopment organelle.

## 4. Sorting of Virions in Polarized and Non-Polarized Cells

### 4.1. The Virally Encoded Membrane Proteins gE, gI, and US9p: An Overview 

Following completion of envelopment, OEVs must be delivered to the cell surface for release and transmission (Figure 2). HSV-1 has evolved to replicate, traffic, and spread within highly polarized cells such as the oral and anogenital mucosa [1,3,22] and their adjacent sensory neurons [5,8,212]. Remarkably, the sorting of HSV-1 particles in these very different kinds of cells shares a common machinery, the gE/gI heterodimer [5,7,22,213] (Table 1, Section 3.6). In the nervous system the sorting functions of gE/gI are in cooperation with US9p, a small (90 amino acids long in HSV-1 strain 17) non-glycosylated lipid raft-associated type II membrane protein, with its carboxy terminal ~20 amino acids imbedded in the lipid bilayer and the amino terminus of the protein directed towards the cytoplasm or the viral tegument (Figure 1) [22,97,98,162,214]. The cytoplasmic tails of gE/gI and US9p contain a number of candidate intracellular sorting signals including acidic clusters, and tyrosine and dileucine motifs [84] that mediate their endocytosis and/or delivery to the TGN [87,151,159,163] (Section 3.3). US9p is usually considered to only participate in HSV-1 sorting within neurons; however, genetic studies hint at a possible role for US9p in spread between Vero cells [52] (Section 4.2). 

### 4.2. Sorting of HSV-1 in Polarized and Non-Polarized Epithelial Cells

In cultured polarized epithelial cells gE/gI are required for the efficient sorting of HSV-1 virions away from apical and towards lateral surfaces, delivering virions to cell–cell junctions to promote efficient spread [22,150,164]. Loss of gE/gI, or certain mutations within them, lead to HSV-1 missorting to apical surfaces and its secretion into the medium, reduced delivery to cell–cell junctions, and compromised cell-to-cell spread [22]. This results in a small plaque phenotype in culture and markedly reduced spread of HSV-1 gE/gI nulls in corneal epithelia [22,215]. In addition to increasing the efficiency of the spread, gE/gI-directed sorting presumably also reduces the exposure of HSV-1 particles to the immune system [22,216]. The mechanism by which gE/gI controls HSV-1 intracellular trafficking in epithelia is not well understood, but one possibility is that the heterodimer ensures delivery of enveloped HSV-1 particles to subdomains of the TGN specialized for sorting [22,217], or even contributes to the selection of the TGN as the envelopment organelle (Section 3.3). In addition, colocalization of gE/gI at lateral surfaces with the adherens junction protein β-catenin suggests that gE/gI might bind extracellular ligands that are concentrated at epithelial cell junctions [88,218]. This could facilitate transfer of HSV-1 particles into the space between cells, movement across the junction, or even localized fusion between infected and uninfected cell membranes [150,218,219]. Such mechanisms are consistent with the finding that mutations in the gE extracellular domain can reduce HSV-1 spread between human HaCaT keratinocytes to levels seen in a gE-null virus, without affecting gE expression, dimerization with gI, or gE/gI incorporation into viral particles [88,164]. The extracellular ligands with which the cell-surface domains of gE/gI would interact to promote HSV-1 spread at cell–cell junctions remain to be identified. 

Several other HSV-1 gene products have been implicated in cell–cell spread including the tegument protein complex UL11p/UL16p/UL21p [48,51] (Section 3.5 and Section 3.6) and the tegument protein and possible ESCRT-III mimic UL51p (Section 3.6 and Section 3.7) [68,69]. There is evidence that each of these molecules affect cell–cell spread, at least in part, via effects on gE/gI activity or trafficking. UL11p binds directly to the gE cytoplasmic tail (Figure 1, Section 3.6) and the UL11p/UL16p/UL21p complex is required for proper gE trafficking to cell junctions [51,68,77]. UL51p also appears to interact with gE and modulates gE localization, or that of other components of the spread machinery [68]. However, UL51-null mutants have a more severe spread defect than gE nulls [68] suggesting additional functions for UL51p, and indeed the UL7p/UL51p complex localizes to, and stabilizes, focal adhesions during HSV-1 infection [67]. This reduces the extent to which cells round-up during the course of infection, and presumably helps maintain cell–cell contacts that are important for viral spread [67]. This role for UL51p appears to be distinct from its interactions with gE, since gE was absent from focal adhesions and was not required for the recruitment of UL7p-UL51p to them [67]. Importantly, the contributions of UL51p to spread are genetically separable from its role in envelopment (Section 3.7), since partial deletion of UL51 resulted in a ~100-fold reduction in plaque size that could not be accounted for by defects in infectious particle production [68]. 

It is increasingly clear that other pathways of HSV-1 sorting exist to help mediate the spread of infection [68]. Even in non-polarized Vero cells, where HSV-1 cell–cell spread is not affected by loss of gE [52], virions are released at specific pocket-like areas of the plasma membrane along the substrate-adherent and cell–cell-adherent surfaces. These virion release sites are heavily enriched in viral glycoproteins and their formation or maintenance is dependent upon the integrity of the actin cytoskeleton [220]. In addition, Vero cell–cell spread was, unexpectedly, dramatically inhibited by a point mutation in the UL34p membrane-anchored subunit of the HSV-1 nuclear export complex, which mediates egress of capsids across the nuclear envelope [12,15,52] (Section 2). Equally surprisingly, UL34p expression was required for proper cell-surface localization of gE, which (as indicated above) is thought to play no role in the spread in Vero cells [52]. These data were taken to suggest that UL34p might be perturbing the expression of multiple viral cell-surface proteins, including some that play a role in an uncharacterized spread pathway [52]. Remarkably, extragenic suppressors of the UL34p spread-defective allele mapped to nonsense mutations in US9p (Section 4.1) [52]. It is unknown whether these US9p mutations repair the original spread defect or uncover additional compensatory pathways of cell–cell HSV-1 transmission, but this work suggests a greater degree of complexity in the relationship between gE/gI, US9p, and HSV-1 sorting and spread in non-neuronal cells than is generally appreciated. 

In addition to playing roles in capsid transport, organelle docking, and envelopment, the inner tegument protein UL37p (Table 1, Section 2, Section 3.2, Section 3.5, Section 3.6 and Section 3.7) may also be important for spread [42]. Several surface regions in the MTC-like UL37Np domain, conserved between PRV and HSV-1 [42,43], are dispensable for PRV propagation yet important for cell–cell spread [42]. This was interpreted to suggest that the MTC-like region of UL37p may help sort intracellular alphaherpesviruses to cell junctions. However, the effects of UL37p upon spread are likely to be manifold; loss of UL37p delays trafficking of PRV capsids to the nucleus in epithelial cells [221], and the UL37p carboxy-terminal domain binding partner dystonin/BPAG1 [127,128] (Section 3.2) influences the efficiency of HSV-1 egress [128] and entry [171]. 

### 4.3. Sorting of HSV-1 in Neurons, the Married and Separate Models

**Figure 3:**
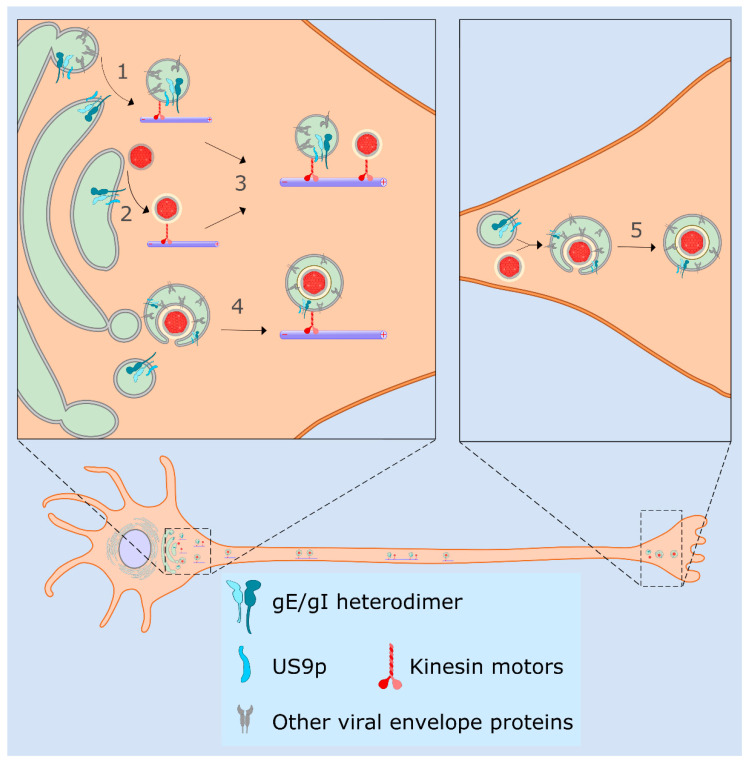
Models for gE/gI-US9p-mediated sorting of HSV-1 during egress in neurons. In the “separate” model, transport vesicles carrying envelope protein cargo recruit kinesin motors using a gE/gI and/or US9p-dependent mechanism (1). Similarly, gE/gI and US9p “load” kinesin motors onto the surface of the HSV-1 capsid shell (2). Since gE/gI and US9p are imbedded in lipid bilayers this process is hypothesized to occur on the cytoplasmic face of an organelle (2), but the mechanism is not well understood. Capsids and transport vesicles then utilize these motors to traffic along cell body and axonal MTs (3) to the nerve terminal, and come together during capsid envelopment to generate the OEV (5). In the “married” model, HSV-1 capsids envelope at cytoplasmic organelles in the neuronal cell body (4) to generate the OEV, much as in non-neuronal cells (Figure 2). gE/gI and US9p may help drive this envelopment and/or subsequently function as membrane-bound receptors to recruit kinesins to the surface of the OEV. The OEV then traffics into and along the axon to transport its cargo of enveloped HSV-1 particles to the nerve terminal. The structures of gE/gI and US9p are represented at the bottom of the figure. Other viral and cellular components are as shown in Figure 1 and Figure 2. See text for more details.

The gE/gI complex modulates trafficking of HSV-1 particles within neurons in cooperation with a third molecule, the membrane protein encoded by US9 (Section 4.1). gE/gI and US9p are required for transport of HSV-1 capsids or enveloped virions from the neuronal cell body into, or along the axon, and are critical for anterograde spread of HSV-1 within the nervous system (Figure 3) [5,7,215,222,223,224]. It appears that gE/gI and US9p function in a largely redundant manner; loss of US9p reduces delivery of HSV-1 particles to distal axons by about 50% [84,86] but simultaneous loss of US9p and gE/gI abolishes it almost completely [85]. Cooperation between gE/gI and US9p is also important for the axonal delivery or trafficking of transport vesicles carrying HSV-1 envelope proteins from the cell body to the nerve terminal [86,225]. 

Understanding the molecular functions of gE/gI and US9p in HSV-1 trafficking and sorting within neurons is complicated by uncertainty concerning the site at which HSV-1 capsids undergo cytoplasmic envelopment [226,227,228] (Figure 3). In one scenario, capsid envelopment takes place in the neuronal cell body; thus, OEVs must be sorted into the axon and trafficked along it to the nerve terminal for release to adjacent epithelial cells. This is termed the “married” model (Figure 3) since HSV-1 capsids, the envelope, and envelope proteins all traffic down the axon as part of the same particle. An alternative suggestion is that HSV-1 capsids travel from the cell body into the axon, traffic along the axon, and undergo envelopment at a distal site such as the nerve cell terminus [5,226,227,228]. This is termed the “separate” model (Figure 3), because capsids and envelope proteins travel separately along the axon, the latter in some sort of carrier transport vesicle. Data exist in support of both the married and separate models [7,84,207,226,227,229,230] and this question has recently been reviewed in detail [5].

Support for the separate mode of HSV-1 transport has come from study of infected explanted human fetal DRGs, where non-enveloped HSV-1 capsids were frequently observed in axons and partially and fully enveloped capsids seen in axonal varicosities and growth cones [231,232,233]. Axonal transport of non-enveloped capsids was also reported in HSV-1-infected rat DRGs, rat TGs, and embryonic rat hippocampal neurons. Similar conclusions were reached for HSV-1 trafficking in cultured human SK-N-SH neuroblastomas [207,234,235] and in the axons of murine retinal ganglia following HSV-1 intraocular infection [225]. In contrast, studies of HSV-1-infected murine [122], rat and chick DRGs [236,237], and infected cultured mouse CAD cells (a derivative of a catecholaminergic central nervous system cell line [238,239]) concluded that HSV-1 traffics within axons as enveloped particles in accord with the married model. Data obtained from infected rat superior cervical ganglia (SCGs) support both models, one study reporting only completely assembled enveloped HSV-1 virions within axons [229], but another finding a 3:1 mixture of OEVs and non-enveloped capsids [240]. 

One way to reconcile these findings is that HSV-1 may have the capacity to utilize both the separate and married transport mechanisms to varying extents, depending upon the origin of the neurons being studied and perhaps the conditions of their culture [84,230,240]. In this case, the ability of non-enveloped capsids and/or OEVs to enter the axon would reflect differences in the gating properties of the axon initial segment (AIS) or pre-axonal filtering zone [241,242,243] that control access of neuronal cell body cargo to the axon. HSV-1 capsids can certainly recruit kinesin motors prior to their envelopment (Section 3.2), so it is conceivable that a poorly established axonal filter would allow both OEVs and non-enveloped capsids to traffic into and along the axon. An alternative explanation for the presence of non-enveloped HSV-1 capsids in axons is that they represent incoming virions that have infected the axon or axon terminal, left their envelope behind at the cell surface, and are undergoing retrograde traffic to the cell body [244]. A more prosaic possibility is that axonal capsids appear non-enveloped in immunocytochemical studies because tegument or envelope proteins are not readily accessible to antibodies in the crowded interior of the axon or virion. Fluorescent protein fusions to envelope proteins provide an alternative means to test whether axonal capsids are associated with their envelopes, but this approach necessitates use of recombinant proteins and viruses. Many of these issues have been discussed in detail elsewhere [7,207,226,227,229,237,244].

### 4.4. Molecular Functions for gE/gI and US9p in the Married and Separate Mechanisms

What functions might gE/gI and US9p serve in the context of these two models? In the “married” model, an appealing possibility is that gE/gI and US9p recruit kinesin motors to the surface of the organelles transporting enveloped virions (Figure 3). This hypothesis is based on a wealth of biochemical, genetic, and imaging data suggesting that PRV gE/gI-US9p functions as a complex to recruit the kinesin-3 motor KIF1A to enveloped PRV particles for delivery into, or trafficking along, axons [5,180,245,246,247,248]. However, in the case of HSV-1, the US9p protein has been reported to interact with the cytoplasmic tail of the kinesin-1 KIF5B [100], and HSV-1 particles appear to utilize KIF5 family members (rather than KIF1A) for transport within the axon [249], complicating comparisons with PRV. An alternative suggestion is that gE/gI and US9p participate in the envelopment of HSV-1 capsids in the cell body (Section 3.6) since an HSV-1 strain simultaneously deleted for gE and US9p accumulated greater numbers of partially enveloped capsids than wild-type controls in the cell bodies of several types of explanted and cultured neurons [84]. Fewer enveloped particles available for transport would thus diminish the numbers of virions seen in axons, particularly as partially enveloped HSV-1 capsids are blocked in their ability to traffic along MTs [197] (Section 3.8). 

Within the context of the separate model it is straightforward to imagine how gE/gI and US9p could regulate the axonal sorting/trafficking of vesicle-associated envelope proteins [86,225]. However, it is much more challenging to envision how the membrane proteins gE/gI and US9p might affect the transport of non-enveloped HSV-1 capsids into or along axons [84]. One hypothesis is that gE/gI and US9p provide “loading” sites on the cytoplasmic surfaces of cell body organelles that bind capsids, attach them to motors, and direct them to axonal MTs for subsequent transport [85,86] (Figure 3). However, the molecular details of such a mechanism remain to be established. Further complicating matters US9p (but not other virally encoded membrane proteins) has been reported to localize with non-enveloped HSV-1 capsids trafficking in axons [86,225]. Perhaps the “non-enveloped” trafficking capsids postulated by the separate model are in fact docked to the cytoplasmic face of an anterograde-trafficking vesicle carrier that contains membrane-anchored US9p and has the capacity to recruit motors [86]. This “hitch-hiking” capsid would ride the vesicle to the nerve terminal then presumably depart from its carrier to engage the envelopment machinery. Membrane-associated but non-enveloped HSV-1 capsids have been observed in axons [7,231], but US9p-positive HSV-1 capsids did not appear to be membrane associated in the axons of mouse retinal ganglia [225]. Finally, US9p has been proposed to facilitate HSV-1 capsid envelopment in neuronal growth cones and varicosities [99], and thus in the context of the separate model plays successive roles in HSV-1 non-enveloped capsid axonal transport then enveloped particle assembly at the nerve terminal. This is interesting in light of the suggestion that gE/gI and US9p perform a similar envelopment function in the neuronal cell body [84] (Section 3.6 and discussed above). An intriguing possibility is that crosstalk between populations of gE/gI and US9p molecules participating in these different stages might serve to coordinate envelopment, motor recruitment, and trafficking.

### 4.5. HSV-1 Transmission via Tunneling Nanotubes

TNTs are 10–100 μm-long membranous processes that extend between cells and serve to transfer ions, proteins, and organelles [119,120]. They vary in thickness and cytoskeletal composition; TNTs with a diameter less than 0.7 μm usually contain only a filamentous F-actin backbone while thicker ones commonly contain both F-actin and MTs [250,251]. Correspondingly, while all TNTs can contain nonconventional myosins such as myosin Va and/or myosin X, “thick” MT-containing TNTs also harbor kinesin-1 and dynein [250,251]. Many different families of viruses appear to direct the formation of TNTs or TNT-like structures during infection in order to transmit infectious particles or signals to neighboring cells [250]. TNT induction has been reported for HSV-1 [252,253,254] and several other alphaherpesviruses [65,250,251], and in many cases the conserved alphaherpesvirus serine/threonine kinase US3p, known to control MT acetylation and stability (Table 1, Section 3.1) is necessary and sufficient for TNT assembly [64,250,255,256]. TNTs induced by HSV-2 US3p contain both actin and β-tubulin, and their formation requires assembly of filamentous actin and the catalytic kinase activity of US3p [64]. TNTs induced by HSV-1 infection of Vero cells contain non-muscle myosin heavy chain IIA (NMIIA), which appears to be associated with the outer tegument protein UL49p (VP22) [252] (Table 1, Section 3.1 and Section 3.6). During HSV-1 infection VP16-GFP and GFP-VP22 fusion proteins were found in fluorescent puncta within TNTs [252,253], and GFP-VP22 colocalized with NMIIA-containing filaments. It is unclear whether those fluorescent foci represent OEVs traversing the TNTs, or other structures containing GFP-VP22 and VP16-GFP; however, the general myosin inhibitor 2,3-Butanedione monoxime (BDM) inhibited the release of the infectious virus to the media by 20–50 fold [252]. Release of HSV-1 from infected HeLa cells was also inhibited by a dominant negative allele of myosin Va [257], a known component of TNTs [250,251], though in that study the possible involvement of TNTs was not addressed [257]. Interestingly, HSV-1-mediated TNT induction is not limited to epithelial cells; HSV-1 infection induced actin-dependent formation of TNT-like processes in neuronally-differentiated mouse p19 cells [254]. 

Do alphaherpesvirus-induced TNTs serve to position sites of virus release close to the surfaces of uninfected target cells, or do they provide continuous bridges between infected and uninfected cell cytoplasm? PRV-induced or PRV-US3p-induced TNTs were found to contain stabilized (acetylated and detyrosinated) MTs, and to transport both OEVs and mitochondria, but not non-enveloped PRV capsids [65,250,258]. Electron and fluorescence microscopy revealed that the points of contact between TNTs and acceptor cells were enriched in adherens junction proteins β-catenin and E-cadherin, and in some cases there appeared to be cytoplasmic connectivity between the TNT and the acceptor cell [258]. Nevertheless, PRV particles were released from the entire length of the TNT, not just at the TNT/acceptor-cell junction [258]. This suggests that PRV transmission by TNTs is via the release of enveloped virions into the extracellular space followed by fusion of the PRV envelope with the plasma membrane of the uninfected cell, rather than by direct transfer from the interior of the TNT to the neighboring cell cytoplasm [258].

In a superficial sense at least, HSV-1 transport along MT-containing TNTs resembles viral transport along axons or neurites [5] (Section 4.3). It remains a fascinating question whether gE/gI or US9p play roles in sorting of cytoplasmic HSV-1 particles to the TNTs, and/or recruitment of kinesin motors for MT-dependent delivery to the TNT tip.

## 5. Late Exocytic Events: Emergence from the Cell Surface 

Delivery of mature HSV-1 particles to the extracellular space is accomplished by fusion between the plasma membrane and the lipid bilayer surrounding the enveloped virion (Figure 2). It is unclear whether the compartment that fuses with the cell surface is identical to that utilized for capsid envelopment (Section 3.3) or whether enveloped virions traffic from the envelopment organelle to other organelles during their intracellular sorting (Section 4). In infected human fetal DRGs enveloped HSV-1 particles are found within organelles containing the cellular proteins Rab3A, SNAP25 and GAP43 [231,232], suggesting they exit the cell through the same pathway used for delivery of synaptic vesicle proteins to the axon terminus, or for regulated exocytosis of neurotransmitters and hormones [232,259]. Total internal reflection fluorescence (TIRF) microscopy revealed that, in epithelial cells, individual PRV particles travel to the plasma membrane inside small, acidified secretory vesicles that also contain Rab6A, Rab8A, and Rab11A, a similar Rab profile to that seen for vesicles mediating constitutive traffic of viral glycoproteins to the plasma membrane [260,261]. Interestingly, PRV L-particles (Section 3.7) appeared to utilize the same constitutive egress route [260]. PRV-containing vesicles underwent fast, directional transport to the site of exocytosis, which was often close to regions of the plasma membrane that labeled with fluorescently tagged LL5β [261], a PIP3-binding protein that connects CLASPs (Section 3.1) at the plus-end of MTs to the plasma membrane [262]. PRV-containing vesicles remained tightly docked at the site of exocytosis for several seconds until membrane fusion occurred, delivering a single PRV particle to the cell surface [261]. Although it remains unclear whether release from neuronal cells is mechanistically similar, transmission of HSV-1 and PRV from cultured rat SCGs to adjacent epithelial cells also involves single viral particles [263].

## 6. Conclusions

HSV-1 capsid trafficking, envelopment and transport, and sorting of enveloped virions in the infected cell cytoplasm can often seem a process of bewildering complexity, and many fundamental questions concerning the biology of this virus remain unanswered. Nevertheless, we are beginning to understand some of the ways in which viral structural components interface with cellular organelles, MTs, and the protein-trafficking apparatus to accomplish viral assembly and spread. Some remaining key questions are as follows:

- How do HSV-1 capsids recognize and select the lipid bilayer of a specific organelle for their envelopment? How does the reorganization of MTs and the MTOC ensure efficient delivery of non-enveloped capsids to that location?
- If the capsid-bound or TGN-associated UL7p/(UL51p)_2_ complex triggers ESCRT-III assembly, how does it choose the location and timing of filament polymerization? How is this function coordinated with the activity of other viral structural proteins known to be important for envelopment?
- What mechanisms exist to ensure that all necessary envelope and outer tegument proteins are loaded into the nascent envelopment site before ESCRT-III-driven envelope scission, the “point of no return”, occurs? How is MT-directed motility of the envelopment intermediate suppressed during assembly?
- How do gE/gI and US9p interact with kinesin motors during egress of capsids and enveloped virions? Which motors are utilized before and after envelopment, and how are they recruited and exchanged? Does motor recruitment and modification of cell–cell junctions explain all of the features of HSV-1 particle sorting in epithelial cells, or do gE/gI serve other functions?

Answers to the above questions are certain to open up exciting new avenues of research into HSV-1 biology, and likely lead to new mysteries. Moreover, given the complex dance between HSV-1 particles, their structural components, and the host cell, we are also confident this virus has much to teach us about organelle biology, intracellular protein transport, and the regulation of molecular motors and MTs in neurons and other cell types.

## Figures and Tables

**Table 1:** HSV-1 structural proteins and complexes discussed in this review.

Protein or Complex (Alternate Names)	Description and Location of Protein(s)	Functions ^1^	Section ^2^ (Key References)
UL11p/UL16p/UL21p	Complex of tegument proteins localized to TGN.	UL11p is palmitoylated and myristoylated. Complex binds to cytoplasmic tail of gE. Envelopment.	3.5, 3.6, 4.2 [45,46,47,48,49,50,51]
UL17p/UL25p	Capsid penton-associated complex. Forms part of the CVSC.	Anchors UL36p to the capsid as a UL17p/(UL25p)_2_ complex. Interacts with VP13/14.	2 [21,24,31]
UL18p/UL38p (VP23/VP19C)	Capsid proteins.	Form VP19c/(VP23)_2_ triplexes that connect VP5 capsomeres.	2 [11,14,20,21]
UL19p (VP5)	Major capsid protein.	Forms penton and hexon capsomeres.	2 [14,21]
UL34p	Type II membrane protein.	Expression influences cell–cell spread. Component of the UL31p/UL34p nuclear export complex.	4.2 [12,16,52]
UL36p (VP1/2)	Inner tegument protein.	Foundation for recruitment of outer tegument via VP16. Binds UL37p. Cooperates with UL37p to recruit kinesin-1 and kinesin-2 to capsid. Envelopment.	2, 3.2, 3.3, 3.5, 3.6, 3.7 [53,54,55,56,57,58,59]
UL37p	Inner tegument protein.	Binds UL36p, dystonin and gK/UL20p. Membrane-tethering (possible mimic of cellular MTCs). Cooperates with UL36p to recruit kinesin-1 and kinesin-2 to the capsid. Envelopment.	2, 3.2, 3.3, 3.5, 4.2 [42,43,44,56,60,61,62]
US3p	Serine/threonine kinase. Inner tegument protein.	MT stabilization and acetylation. Assembly of TNTs.	2, 3.1, 4.5 [26,28,63,64,65]
UL7p/UL51p	Outer tegument protein complex.	May mimic or trigger assembly of cellular ESCRT-III complex. Envelopment. Cell–cell spread. Localizes to focal adhesions.	3.6, 3.7, 4.2 [66,67,68,69]
UL46p (VP11/12)	Outer tegument protein.	Binds UL48p (VP16).	3.6 [11]
UL47p (VP13/14)	Outer tegument protein.	Binds UL48p (VP16). Binds CVSC component UL17p. Envelopment.	3.6 [11,70]
UL48p (VP16)	Outer tegument protein.	Connects UL36p to outer tegument. Binds gH carboxy-terminal tail. Envelopment.	2, 3.6 [11,34,35,36,71,72,73]
UL49p (VP22)	Outer tegument protein.	MT acetylation, bundling and stabilization. Binds UL48p (VP16), gD, gE, gM. Envelopment.	2, 3.1, 3.6 [11,74,75,76,77]
gB	Type I membrane protein.	Loss of gB and gD reduces envelopment. Required for fusion ^3^.	3.3, 3.4, 3.6 [78,79,80,81,82]
gD	Type I membrane protein.	Loss of gD and gB or gE/gI reduces envelopment. Binds VP22. Required for fusion ^3^.	3.4, 3.6 [80,81,83]
gE/gI	Heterodimer of type I membrane proteins.	Loss of gE/gI and gD disrupts envelopment. Sorting of virions to epithelial junctions and into or along axons. Loss of gE/gI and US9p reduces envelopment in neurons. gE binds VP22 and the UL11p/UL16p/UL21p complex.	3.6, 4 [77,83,84,85,86,87,88,89]
gH/gL	Heterodimer of type I membrane protein (gH) with lumenal/extracellular soluble subunit (gL).	Required for fusion ^3^. Binds UL48p (VP16).	3.4, 3.6 [35,36,81,90]
gK/UL20p	Heterodimer of multi membrane- spanning proteins.	Regulation of gB/gD/gH/gL-mediated fusion ^3^. Binds UL37p. Sorting of gD and gH/gL to envelopment site. Envelopment.	3.4, 3.5, 3.6 [61,90,91,92,93]
gM gM/gN	Multi membrane- spanning protein (gM). Can form complex with type 1 membrane protein gN	Sorting of gD and gH/gL to envelopment site. Envelopment.	3.4, 3.6 [90,94,95,96]
US9p	Type II membrane protein.	Loss of US9p and gE/gI reduces envelopment in neurons. Sorting of virions into or along axons.	4 [97,98,99,100]

^1^ Known functions relevant to this review. ^2^ Section(s) of this review where the protein/complex is discussed. ^3^ Fusion between HSV-1 envelope, or infected cell surface, and uninfected cell.

## Abbreviations

HSV-1	Herpes simplex virus type 1
PRV	Pseudorabies virus (suid alphaherpesvirus 1)
ULnumber	Unique long (position of gene in HSV-1 genome)
USnumber	Unique short (position of gene in HSV-1 genome)
VPnumber	Virus protein
gletter.	Virally encoded membrane glycoprotein
ER	Endoplasmic reticulum
ESCRT	Endosomal sorting complex required for transport
MT	Microtubules
OEV	Organelle-associated enveloped virion
TGN	Trans-Golgi network
TNT	Tunneling nanotube
